# Neutrophilic noncoding RNAs predict outcomes of acute ischemic stroke patients treated with recombinant tissue plasminogen activator

**DOI:** 10.3389/fphar.2022.1003806

**Published:** 2022-10-06

**Authors:** Ziping Han, Lingzhi Li, Zhen Tao, Rongliang Wang, Haiping Zhao, Yangmin Zheng, Zhenhong Yang, Liyuan Zhong, Junfen Fan, Yumin Luo

**Affiliations:** ^1^ Institute of Cerebrovascular Diseases Research and Department of Neurology, Xuanwu Hospital of Capital Medical University, Beijing, China; ^2^ Beijing Key Laboratory of Translational Medicine for Cerebrovascular Diseases, Beijing, China; ^3^ Beijing Institute for Brain Disorders, Beijing, China

**Keywords:** acute ischemic stroke, neutrophil, noncoding RNA, outcome, recombinant tissue plasminogen activator, symptomatic intracerebral hemorrhage

## Abstract

There’s no evidence demonstrating the association between noncoding RNAs levels before IV recombinant tissue plasminogen activator (rtPA) administration and the outcomes of acute ischemic stroke (AIS). 145 AIS patients received rtPA treatment were recruited at the stroke center from 2018 to 2019, and 103 patients were included in this study. A panel of noncoding RNAs (miRNA-23a, miRNA-193a, miRNA-128, miRNA-99a, miRNA-let-7a, miRNA-494, miRNA-424, and lncRNA H19) were measured in the circulating neutrophils of AIS patients before rtPA treatment. Endpoints included excellent outcome (modified Rankin Scale score [mRS] 0–1) or poor outcome (mRS > 1) at 3 months and symptomatic intracerebral hemorrhage (sICH) after rtPA treatment. Among the eight noncoding RNAs detected in circulating neutrophils of the 103 participants, miRNA-23a levels were associated with the stroke severity on admission and symptom progression at 24 h after rtPA treatment. A noncoding RNA score composed of miRNA-23a, miRNA-99a, and lncRNA H19 was screened to predict the functional outcome at 3 months and the incidence of sICH after rtPA treatment. In the logistic regression analysis, the noncoding RNA score ≥ −0.336 (OR = 2.862 [1.029–7.958], *p* = 0.044) was an independent predictor of the poor outcome at 3 months after adjustment of clinical variables, the addition of the noncoding RNA score to the clinical model improved the discrimination (IDI% = 4.68 [0.65–8.71], *p* = 0.020), as well as the net reclassification (NRI% = 33.04 [0.54–71.49], *p* = 0.016). The noncoding RNA score ≥ −0.336 (OR = 5.250 [1.096–25.135], *p* = 0.038) was also independently predicted the sICH, the addition of the noncoding RNA score to the clinical variables improved discrimination and reclassification as well. The noncoding RNA score was also associated with the infarct volume and symptom improvement at 7 days after rtPA treatment. In conclusion, a higher neutrophilic noncoding RNA score provides predictive value to identify AIS patients with worse outcomes after rtPA treatment. miRNA-23a, miRNA-99a, and lncRNA H19 are worth further investigation for their effects in thrombolysis after AIS.

## Introduction

Recombinant tissue plasminogen activator (rtPA) is the standard thrombolytic agent approved for recanalization to salvage the penumbra after acute ischemic stroke (AIS) (Powers et al., 2019). Ideally, it can effectively dissolve the thrombus and ensure the blood reflows to the ischemic brain tissue. In the real world, however, some AIS patients could not achieve recanalization, some achieve futile recanalization, and some suffer from hemorrhage transformation and malignant cerebral edema, resultantly only 5–10% of AIS patients benefit from rtPA treatment (Wardlaw et al., 2012; Zerna et al., 2018). Thereby, searching for biomarkers to predict the outcome after rtPA treatment can help identify patients with poor responses to thrombolysis and provide opportunities for bridging therapy.

Noncoding RNA (ncRNA) is one of the most potential biomarkers for diseases (Karakas and Zeller, 2017), and lines of studies have confirmed the key role of ncRNA in the pathological processes of ischemic stroke (Dykstra-Aiello et al., 2016; Li et al., 2018a). Preclinical studies in the experimental stroke model indicated that miRNA-23a (Zhao et al., 2014) and miRNA-424 (Liu et al., 2015) lessened cerebral ischemia-reperfusion injury by inhibiting oxidative stress; miRNA-99a (Tao et al., 2015) and miRNA-128 (Liu et al., 2019) could prevent neuron apoptosis via regulating cell cycle reentry; miRNA-let-7a was involved in the neuroprotection of remote postconditioning (Vinciguerra et al., 2020); miRNA-494 (Li et al., 2020) and miRNA-193a-5p modulated neuroinflammation by regulating phenotypic transformation of neutrophils; and lncRNA H19 promoted neuroinflammation by driving M1 microglial polarization (Wang et al., 2017) and leukocyte activation (Li et al., 2022a). Furthermore, pieces of clinical studies also showed that miRNA-424 (Li et al., 2018b) and miRNA-99a-5p (Zhao et al., 2017) levels in circulating immune cells and circulating lncRNA H19 (Wang et al., 2017) levels possessed diagnostic potential for ischemic stroke; miRNA-128 levels in circulating lymphocytes of AIS patients were correlated with stroke severity (Liu et al., 2019); and miRNA-494 in circulating neutrophils could predict the clinical outcomes of AIS patients (Li et al., 2020). However, there’s no evidence demonstrating the association between these noncoding RNAs before rtPA administration and the outcomes of AIS.

The aim of this study, therefore, was to investigate the association between the eight potential noncoding RNA candidates (miRNA-23a, miRNA-193a, miRNA-128, miRNA-99a, miRNA-let-7a, miRNA-494, miRNA-424, and lncRNA H19) in the circulating neutrophils of AIS patients before rtPA treatment and stroke outcomes as assessed by symptomatic intracerebral hemorrhage (sICH), and modified Rankin Scale (mRS) at 3 months.

## Materials and methods

### Study design and participants

From November 2018 to May 2019, 345 AIS patients were enrolled in the stroke center of Xuanwu Hospital of Capital Medical University. A total of 145 patients received intravenous thrombolysis with rt-PA. The inclusion criteria were as follows: age ≥ 18 years old; patients presented with neurological deficits and admission within 4.5 h after symptoms onset; a confirmed diagnosis of ischemic stroke (Powers et al., 2019) by brain magnetic resonance imaging (MRI) or computed tomography (CT); and clinical evaluation conducted and recorded at 3 months post-stroke. The exclusion criteria included: patients with TIA, cerebral haemorrhage, epilepsy, or other nonischemic neurological disease; patients with tumors, heart failure, renal failure, hepatic dysfunction, rheumatic-immune systemic diseases, hematological disorders, active infection, and others; treatment with anti-inflammatory drugs within 1 month before admission; and patients received bridging therapy. Finally, 103 AIS patients received rtPA treatment were enrolled in our analysis (Supplementary Figure E1).

The primary outcome was defined as an excellent outcome (modified Rankin scale [mRS] score (van Swieten et al., 1988), 0–1) and poor outcome (mRS > 1) at 3 months after stroke, and the secondary outcome was sICH after stroke defined according to the ECASS-III (Neuberger et al., 2017).

### Clinical assessments

Baseline variables included demographic characteristics, stroke characteristics and treatment, medical history, stroke etiology, lesion location, and clinical and laboratory findings collected from clinical interviews and neurologic examinations by board-certified neurologists. Stroke severity was assessed by the NIH Stroke Scale (NIHSS) (Lyden et al., 1994) on admission in all enrolled patients. Stroke etiology was defined according to the Trial of ORG 10172 in Acute Stroke Treatment (TOAST) (Adams et al., 1993) classification. NIHSS scores at 24h and 7 days after rtPA treatment were also recorded, 24 hΔNIHSS (NIHSS_baseline_ - NIHSS_24h_) and 7dΔNIHSS (NIHSS_baseline_ - NIHSS_7days_) were then recorded (ΔNIHSS ≥ 4 was considered as an improvement of symptoms). The functional outcome was evaluated with the mRS via the face-to-face visit or the telephone interview at 3 months conducted by trained neurologists. All patients and investigators involved in the present study were blinded to the noncoding RNA measures.

### Imaging

All patients underwent either a non-contrast CT or MRI scan in the emergency room. Patients who received rtPA treatment underwent another CT or MRI scan 24–36 h after treatment, or earlier in case of clinical worsening. A subgroup of patients underwent MRI within 24 h of admission (*n* = 72), and infarct volumes were calculated on DWI sequences as described previously (Wu et al., 2020).

### Intravenous thrombolysis administration

IV rtPA (Actilyse; Boehringer, Ingelheim, Germany) administration was conducted according to the recommendations of the The European Stroke Organisation (ESO) and Executive Committee and the ESO (2008).

### Noncoding RNAs measurements

Circulating neutrophils were obtained from the blood sampled before rtPA treatment in all patients, preserved in TRIzol Reagent (Invitrogen, Cat#15596026) and then stored in the −80°C refrigerator until detection. All samples were handled according to the same protocol throughout the study.

Total RNA was extracted from the neutrophil samples with TRIzol Reagent, and then quantified with nanodrop. According to the manufacturer’s instructions, 1 μg of total RNA in a final volume of 20 μl was used for miRNA First Strand cDNA Synthesis (Tailing Reaction) (Sangon Biotech, Cat#B532451) and lncRNA reverse transcription was conducted with a Hifair^®^ Ⅱ 1st Strand cDNA Synthesis Kit (Yeasen, Shanghai, China, Cat#11121ES60). Total cDNA was followingly prepared for RT-qPCR with the Hieff^®^ qPCR SYBR Green Master Mix (Yeasen, Shanghai, China, Cat#11202ES08) in a QuantStudio^™^ Real-time PCR System (Applied Biosystems, United States). The expression of universal U6 was used as the control for miRNA, and GAPDH was used as the control for lncRNA. 2^−ΔΔCT^ method was used for relative quantification of noncoding RNA expression. Each quantitative PCR assay was performed in triplicate independently. The sequences of the primers used in the present study were listed in file e1. All samples were tested by a professional technician blinded to the clinical assessments.

### Statistical analysis

All the data were analyzed with SPSS 26 (SPSS Inc., Chicago, IL), R studio 3.6.1 (Boston, MA), and GraphPad Prism 8.4.0 (GraphPad Software, La Jolla, CA, United States). *p* < 0.05 was considered of statistical significance.

In the first step, we compared the baseline characteristics and noncoding RNAs levels of the patients according to the stroke severity on admission (Table 1). Specifically, we described the continuous variables as the means (SD) in case of a normal distribution or as the medians [IQRs] otherwise and described the categorical variables as proportions. According to the nature of data distributions, the groups for continuous variables were analyzed by the Student t-test or Mann-Whitney U test; and the groups for categorical variables were analyzed using the Pearson χ^2^ test or Fisher exact test. We assessed the normality of data distribution with the Kolmogorov-Smirnov test.

**TABLE 1:** Baseline characteristics of the study population according to the stroke severity on admission.

	Total	Mild stroke ^a^	moderate-to-severe stroke	*p* Value
	(N = 103)	(N = 55)	(N = 48)	
Demographic characteristics				
Age, y, mean (SD)	61.9 (12.7)	60.6 (12.1)	63.4 (13.2)	0.266
Female sex (%)	22 (21.4)	9 (16.4)	13 (27.1)	0.279
BMI, kg/m^2^, median [IQR]	25.6 [25.1, 26.2]	25.6 [24.4, 26.0]	25.6 [25.6, 26.3]	0.220
Medical history				
Hypertension	71 (68.9)	36 (65.5)	35 (72.9)	0.547
Diabetes mellitus	29 (28.2)	16 (29.1)	13 (27.1)	0.995
Hyperlipemia	23 (22.3)	10 (18.2)	13 (27.1)	0.398
Coronary heart disease	19 (18.4)	8 (14.5)	11 (22.9)	0.402
Atrial Fibrillation	17 (16.5)	5 (9.1)	12 (25.0)	0.047 †
Recurrent stroke	30 (29.1)	17 (30.9)	13 (27.1)	0.835
Smoking habit	39 (37.9)	21 (38.2)	18 (37.5)	—
Clinical and laboratory findings				
Systolic blood pressure, mm Hg	150.0 [140.0, 166.0]	152.0 [140.0, 169.5]	150.0 [135.8, 161.5]	0.302
Diastolic blood pressure, mm Hg	83.0 [74.5, 92.0]	83.0 [74.5, 93.0]	82.5 [74.8, 91.2]	0.840
Serum glucose, mmol/L	7.1 [5.9, 10.5]	7.0 [5.8, 9.0]	7.4 [6.3, 11.9]	0.081
Neutrophils, ×1,000/mm^3^	4.8 [3.7, 6.3]	4.8 [3.9, 5.9]	4.6 [3.5, 7.7]	0.995
NLR	1.6 [1.2, 2.2]	1.7 [1.3, 2.2]	1.5 [1.1, 2.2]	0.143
Platelet count, ×1,000/mm^3^	2.8 [2.0, 4.6]	2.7 [2.2, 3.6]	2.9 [2.0, 6.1]	0.305
HYC, μmol/L	207.0 [169.5, 240.0]	207.0 [170.5, 261.0]	207.0 [168.5, 232.5]	0.945
TG, mmol/L	1.7 [1.1, 2.7]	1.7 [1.0, 2.8]	1.7 [1.2, 2.5]	0.809
TC, mmol/L	4.6 [3.8, 5.4]	4.6 [3.7, 5.6]	4.5 [3.9, 5.1]	0.786
HDL, mmol/L	1.2 [1.0, 1.4]	1.2 [1.0, 1.4]	1.1 [1.0, 1.3]	0.169
LDL, mmol/L	2.6 [2.1, 3.4]	2.6 [1.9, 3.5]	2.6 [2.1, 3.3]	0.704
Onset-to-treatment time, h	2.2 [1.2, 3.2]	2.0 [1.1, 2.9]	2.7 [1.4, 3.3]	0.042 †
Stroke etiology (TOAST), n (%)				
Large artery atherosclerosis	63 (61.2)	36 (65.5)	27 (56.2)	0.451
Small vessel occlusion	17 (16.5)	12 (21.8)	5 (10.4)	0.197
Cardioembolic	12 (11.7)	3 (5.5)	9 (18.8)	0.073
Other determined	4 (3.9)	1 (1.8)	3 (6.2)	0.516
Undetermined	7 (6.8)	3 (5.5)	4 (8.3)	0.852
Posterior circulation stroke	10 (9.7)	4 (7.3)	6 (12.5)	0.575
Outcome measurements				
24 h NIHSS score	4.0 [2.0, 6.0]	3.0 [1.2, 4.0]	6.0 [4.0, 11.0]	<0.001†
7days NIHSS score	1.0 [1.0, 4.0]	1.0 [0.0, 2.0]	4.0 [1.0, 9.0]	<0.001†
sICH	14 (13.6)	5 (9.1)	9 (18.8)	0.255
Excellent outcome ^b^	62 (60.2)	44 (80.0)	18 (37.5)	<0.001†
Noncoding RNA measures				
miR-23a	2.5 [1.1, 5.7]	1.7 [0.9, 3.8]	3.8 [1.4, 10.0]	0.005 †
miR-193a	2.0 [1.2, 3.4]	1.8 [1.1, 3.1]	2.6 [1.2, 4.5]	0.125
miR-128	3.7 [1.5, 7.1]	3.4 [1.5, 8.5]	3.8 [1.9, 6.5]	0.911
miR-let-7a	3.9 [2.0, 8.4]	3.2 [1.6, 7.7]	5.4 [2.7, 8.9]	0.105
miR-99a	2.3 [1.2, 4.8]	1.9 [1.2, 4.0]	2.8 [1.1, 5.2]	0.431
miR-494	1.2 [0.7, 2.4]	1.2 [0.7, 1.9]	1.2 [0.7, 3.0]	0.431
miR-424	3.6 [2.5, 5.7]	3.4 [2.2, 4.4]	3.9 [2.8, 7.1]	0.117
Lnc H19	1.7 [1.1, 2.8]	1.7 [1.1, 2.6]	1.7 [1.1, 3.0]	0.606

a The mild stroke was defined as a NIHSS, score ≤ 5 and the moderate to severe stroke was defined as a NIHSS, score > 5.

b The excellent outcome was defined as an mRS, score = 0–1.

Abbreviations: BMI, Body Mass Index; HDL, High density lipoprotein; HYC, Homocysteine; LDL, Low density lipoprotein; Lnc = Long noncoding RNA; miR = microRNA; mRS, modified Rankin Scale; NIHSS = NIH, Stroke Scale; NLR, Neutrophil-to-Lymphocyte Ratio; rtPA, recombinant tissue plasminogen activator; sICH, symptomatic intracerebral hemorrhage; TC, Total cholesterol; TG, Triglyceride; TOAST, Trial of ORG, 10172 in Acute Stroke Treatment.

Data for continuous variables are described as mean (SD) (normally distributed variables) or as median [interquartile range] (nonnormally distributed variables), for categorical variables are described as n (%).

† *p* < 0.05.

In the second step, to predict the outcomes based on joint consideration of all eight noncoding RNAs simultaneously and reduce possible overfitting, we constructed the noncoding RNA score with a penalized regression approach (Burke Quinlan et al., 2015). In detail, the least absolute shrinkage and selection operator (LASSO) regression was chosen to select a subset of useful predictors to construct the noncoding RNA score from the eight candidates, through five-fold cross-validation. LASSO regression was first applied to the 103 AIS patients and the analysis was performed according to the functional outcome at 3 months. The scores were defined as: Noncoding RNA Score = *β*_0_ + *β*_A *_ noncoding RNA_A_ + *β*_B *_ noncoding RNA_B_ + *β*_c *_ noncoding RNA_C_, etc., where β_A_, β_B_, β_c,_ etc represented regression β-coefficients for noncoding RNA _A,_ noncoding RNA _B,_ and noncoding RNA _C,_ respectively, and β_0_ represented an intercept from the LASSO regression. The noncoding RNA score was also applied to the analysis of the secondary outcome of the patients.

In the third step, the predictive value of the noncoding RNA score for stroke outcomes was calculated with the receiver operating characteristics (ROC) curve. The noncoding RNA score was cut off by the index Youden (1950) into dichotomous variables. Then, we assessed the association of the dichotomous noncoding RNA score with stroke outcomes by multivariate logistic regression analysis. We entered baseline clinical variables (age, admission NIHSS score, a history of hypertension, coronary heart disease, atrial fibrillation, cardioembolic stroke, onset-to-treatment time, neutrophil-to-lymphocyte ratio, and total cholesterol) as confounders to the multivariate analysis for the functional outcome at 3 months of patients received rtPA treatment; and enter age, sex, admission NIHSS score, a history of hypertension, atrial fibrillation, cardioembolic stroke, onset-to-treatment time, and low-density lipoprotein as confounders to the multivariate analysis for sICH. Adjusted odds ratios (ORs) and corresponding 95% confidence intervals (CIs) for each variable were calculated in the models. Improvement of discrimination and reclassification of the predictive models with the addition of noncoding RNA score was calculated by the integrated discrimination improvement (IDI) and categorical or continuous net reclassification improvement (NRI) indexes (Pencina et al., 2010).

### Bioinformatic analysis

The prediction of the mRNA targets of the noncoding RNAs was performed based on Starbase. Gene Ontology (GO) and Kyoto Encyclopedia of Genes and Genome (KEGG) enrichment analysis of the noncoding RNAs in the noncoding RNA score was conducted using the Database for Annotation, Visualization, and Integrated Discovery (DAVID). *p* < 0.05 was set as the criterion for statistical significance.

### Standard protocol approvals, registrations, and patient consents

The study design was complied with the principles of the Declaration of Helsinki, and approved by the Ethics Committee of Xuanwu Hospital, Capital Medical University. Written, informed consents were obtained from all enrolled patients or their legal representatives.

## Results

### Patient characteristics and clinical variables

The baseline clinical characteristics of the 103 AIS patients received rtPA treatment were listed in Table 1. The mean ± SD age was 61.9 ± 12.7 years, and 22 (21.4%) of the patients were female. The median NIHSS score was 5 [IQR 3–11] on admission, the median time from stroke onset to treatment was 2.2 [1.2, 3.2] hours, 10 (9.7%) of the patients presented with posterior circulation stroke, and 63 (61.2%) of the patients presented with large artery atherosclerosis stroke. 55 (53.4%) of the patients presented with mild stroke (NIHSS ≤ 5) and 48 (46.6%) of the patients presented with moderate-to-severe stroke (NIHSS > 5, Table 1). Among the demographic characteristics, only a lower proportion of atrial fibrillation (9.1 vs. 25.0%, *p* = 0.047, for patients with mild stroke and moderate-to-severe stroke, respectively), and a less onset-to-treatment time (2.0 [1.1, 2.9] vs. 2.7 [1.4, 3.3], *p* = 0.042) were associated with the mild stroke on admission (Table 1).

Among the outcome measurements, the median NIHSS score was 4.0 [2.0, 6.0] 24 h after admission, the median NIHSS score was 1.0 [1.0, 4.0] at 7 days after admission, 14 (13.6%) of the patients had symptomatic intracerebral hemorrhage after stroke and 62 (60.2%) of the patients had excellent outcome at 3 months (Table 1).

### Noncoding RNAs in the study population

**FIGURE 1:**
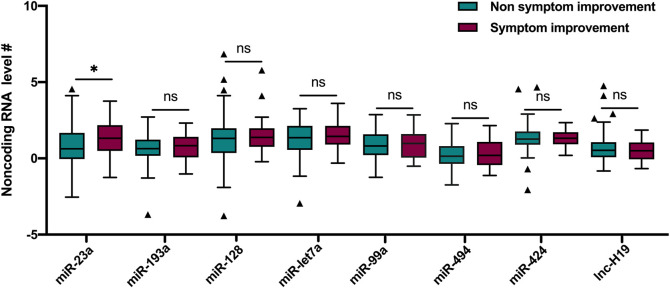
Comparisons of noncoding RNAs between patients with and without symptom improvement at 24 h after rtPA treatment. # Ln transformation of the eight noncoding RNAs. Lines of the boxes represented medians, and error bars represented interquartiles. rtPA, recombinant tissue plasminogen activator. miR, miRNA; lnc, lncRNA.

Among the noncoding RNAs measured in this study, a lower miRNA-23a level (1.7 [0.9, 3.8] vs. 3.8 [1.4, 10.0], *p* = 0.005) was associated with the mild stroke on admission (Table 1). According to the symptom progression at 24 h after rtPA treatment, a higher miRNA-23a level (3.8 [1.8, 8.7] vs. 1.9 [1.0, 5.2], *p* = 0.045) was associated with the 24 h symptom improvement after rtPA treatment (Figure 1).

In the correlation analysis, increased onset-to-treatment time was correlated with decreased levels of miRNA-let-7a (*ρ* = −0.19, *p* < 0.05), miRNA-99a (*ρ* = −0.20, *p* < 0.05), and miRNA-494 (*ρ* = −0.22, *p* < 0.05) (Supplementary Table E1); increased neutrophil count was correlated with decreased miRNA-193a (*ρ* = −0.22, *p* < 0.05) and miRNA-let-7a (*ρ* = −0.20; *p* < 0.05); increased NLR was correlated with decreased miRNA-193a (*ρ* = −0.17, *p* < 0.05) and miRNA-128a (*ρ* = −0.21, *p* < 0.05) (Supplementary Table E1).

### Noncoding RNA score for the excellent outcome of patients treated with recombinant tissue plasminogen activator

To find the association between the noncoding RNAs and outcomes, we constructed the noncoding RNA score using the LASSO regression. miRNA-23a, miRNA-99a, and lncRNA H19 were screened as ideal variables among the eight candidate ncRNAs for the noncoding RNA score. A cutoff point for the noncoding RNA score of −0.336 had 48.8% sensitivity and 69.4% specificity for the prediction of the excellent outcome at 3 months (Supplementary Table E2). Patients with noncoding RNA score ≥ −0.336 (30.6 vs. 48.8%, *p* = 0.009) had a lower incidence of excellent functional outcome at 3 months.

Logistic regression analyses showed that noncoding RNA score ≥ −0.336 (OR = 2.155 [1.053–4.876], *p* = 0.045) was independently predictive of functional outcome at 3 months. After adjustment with clinical variables including age, admission NIHSS score, a history of hypertension, coronary heart disease, atrial fibrillation, cardioembolic stroke, onset-to-treatment time, neutrophil-to-lymphocyte ratio, and total cholesterol (Supplementary Table E3), noncoding RNA score ≥ −0.336 (OR = 2.862 [1.029–7.958], *p* = 0.044) was still an independent predictor of functional outcome at 3 months (Table 2).

**TABLE 2:** Noncoding RNA score and the risk of the outcomes after rtPA treatment (n = 103).

	Model 1*		Model 2^&^	
	OR (95% CI)	*p* value	OR (95% CI)	*p* value
Noncoding RNA score^#^ for excellent outcome	2.155 (1.053–4.876)	0.045†	2.862 (1.029–7.958)	0.044 †
Noncoding RNA score^#^ for sICH	2.495 (0.794–7.838)	0.118	5.250 (1.096–25.135)	0.038 †

Abbreviations: rtPA, recombinant tissue plasminogen activator; sICH, symptomatic intracerebral hemorrhage.

† *p* < 0.05.

#Noncoding RNA, score = 0.017661997*miR-23a-0.092415475*miR-99a-0.007679703*lnc19-0.209855481.

*Model 1 was an unadjusted logistic regression model with the noncoding RNA, score.

&Model 2 was an adjusted logistic regression model. The variables in model 2 for excellent outcome included age, admission NIHSS, score, a history of hypertension, coronary heart disease, atrial fibrillation, cardioembolic stroke, onset-to-treatment time, neutrophil-to-lymphocyte ratio, total cholesterol, and noncoding RNA, score on admission; the variables in model 2 for sICH, included age, sex, admission NIHSS, score, a history of hypertension, atrial fibrillation, cardioembolic stroke, onset-to-treatment time, low-density lipoprotein, and noncoding RNA, score on admission.

The benefit of the addition of the noncoding RNA score for predicting the excellent outcome after rtPA treatment was assessed by the improvement of discrimination and reclassification of the predictive models, adding the noncoding RNA score to the clinical variables improved the integrated discriminatory ability of the clinical predictive model (IDI% = 4.68 [0.65–8.71], *p* = 0.020), as well as the net reclassification (NRI% = 33.04 [0.54–71.49], *p* = 0.016) (Table 3).

**TABLE 3:** Reclassification of the outcomes by noncoding RNA score among AIS patients received rtPA treatment (*n* = 103).

	NRI		IDI	
	Estimate (95% CI),%	*p* value	Estimate (95% CI),%	*p* value
Clinical model for excellent outcome*	Reference	—	Reference	—
Clinical model* + Noncoding RNA score^#^	33.04 (0.54–71.49)	0.016	4.68 (0.65–8.71)	0.020
Clinical model for sICH^&^	Reference	—	Reference	—
Clinical model^&^ + Noncoding RNA score^#^	85.87 (32.87–138.88)	0.002	9.66 (2.07–17.26)	0.010

Abbreviations: IDI, Integrated discrimination improvement; NRI, Net reclassification improvement; rtPA, recombinant tissue plasminogen activator; sICH, symptomatic intracerebral hemorrhage.

#Noncoding RNA, score = 0.017661997*miR-23a-0.092415475*miR-99a-0.007679703*lnc19-0.209855481.

*The clinical model for excellent outcome included age, admission NIHSS, score, a history of hypertension, coronary heart disease, atrial fibrillation, cardioembolic stroke, onset-to-treatment time, neutrophil-to-lymphocyte ratio, and total cholesterol.

&The clinical model for sICH, included age, sex, admission NIHSS, score, a history of hypertension, atrial fibrillation, cardioembolic stroke, onset-to-treatment time, and low-density lipoprotein.

### Noncoding RNA score for the symptomatic intracerebral hemorrhage of patients treated with recombinant tissue plasminogen activator

According to sICH after rtPA treatment, there’re 14 patients with sICH and 89 patients without, the cutoff point for the noncoding RNA score of −0.336 had 51.7% sensitivity and 77.7% specificity for the prediction of the sICH after rtPA treatment (Supplementary Table E2). Logistic regression analyses showed that a noncoding RNA score ≥ −0.336 (OR = 5.250 [1.096–25.135], *p* = 0.038) was an independent predictor of sICH with the adjustment of clinical variables (Table 2).

As the comparisons between the two predictive models shown in Table 3, the addition of the noncoding RNA score to the clinical variables improved the integrated discriminatory ability of the present predictive model (IDI% = 9.66 [2.07–17.26], *p* = 0.010), as well as the net reclassification (NRI% = 85.87 [32.87–138.88], *p* = 0.002).

### Bioinformatic analysis of the noncoding RNA score

A lower noncoding RNA score (−0.4 [−0.6, −0.3] vs.−0.3 [−0.5, −0.3], *p* = 0.038) was associated with the symptom improvement at 7 days after rtPA treatment (Supplementary Table E4), but not with the symptom improvement at 24 h (Supplementary Table E5). In the subgroup of patients (*n* = 72) with available infarct volume, the noncoding RNA score was significantly correlated with the infarct volume (*ρ* = 0.28, *p* = 0.016) (Table 4). Among the three noncoding RNA, a higher neutrophilic miR-23a level was associated with a higher miR-99a level (*ρ* = 0.54, *p* < 0.001), a higher neutrophilic miR-23a level was associated with a higher lncRNA H19 level (*ρ* = 0.31, *p* = 0.002), and a higher neutrophilic miR-99a level was associated with a higher lncRNA H19 level (*ρ* = 0.20, *p* = 0.044) (Table 5).

**TABLE 4:** Bivariate correlation between noncoding RNAs and noncoding RNA score and the infarct volume in the subgroup of patients with available infarct volume (*n* = 72).

	Infarct volume, ml	
	ρ	*p* Value
miR-23a	0.07	0.545
miR-193a	0.07	0.549
miR-128	−0.12	0.306
miR-let7a	0.08	0.481
miR-99a	−0.18	0.141
miR-494	−0.18	0.138
miR-424	−0.06	0.623
Lnc H19	−0.09	0.451
Noncoding RNA score^#^	0.28†	0.016

† *p* < 0.05.

#Noncoding RNA score = 0.017661997*miR-23a-0.092415475*miR-99a-0.007679703*lnc19-0.209855481.

**TABLE 5:** Bivariate correlation between the three noncoding RNA (miRNA-23a, miRNA-99a, and lncRNA H19) in the whole study population (*n* = 103).

	ρ	*p* Value
miR-23a and miR-99a	0.54	< 0.001
miR-23a and lncRNA H19	0.31	0.002
miRNA-99a and lncRNA H19	0.20	0.044

**FIGURE 2:**
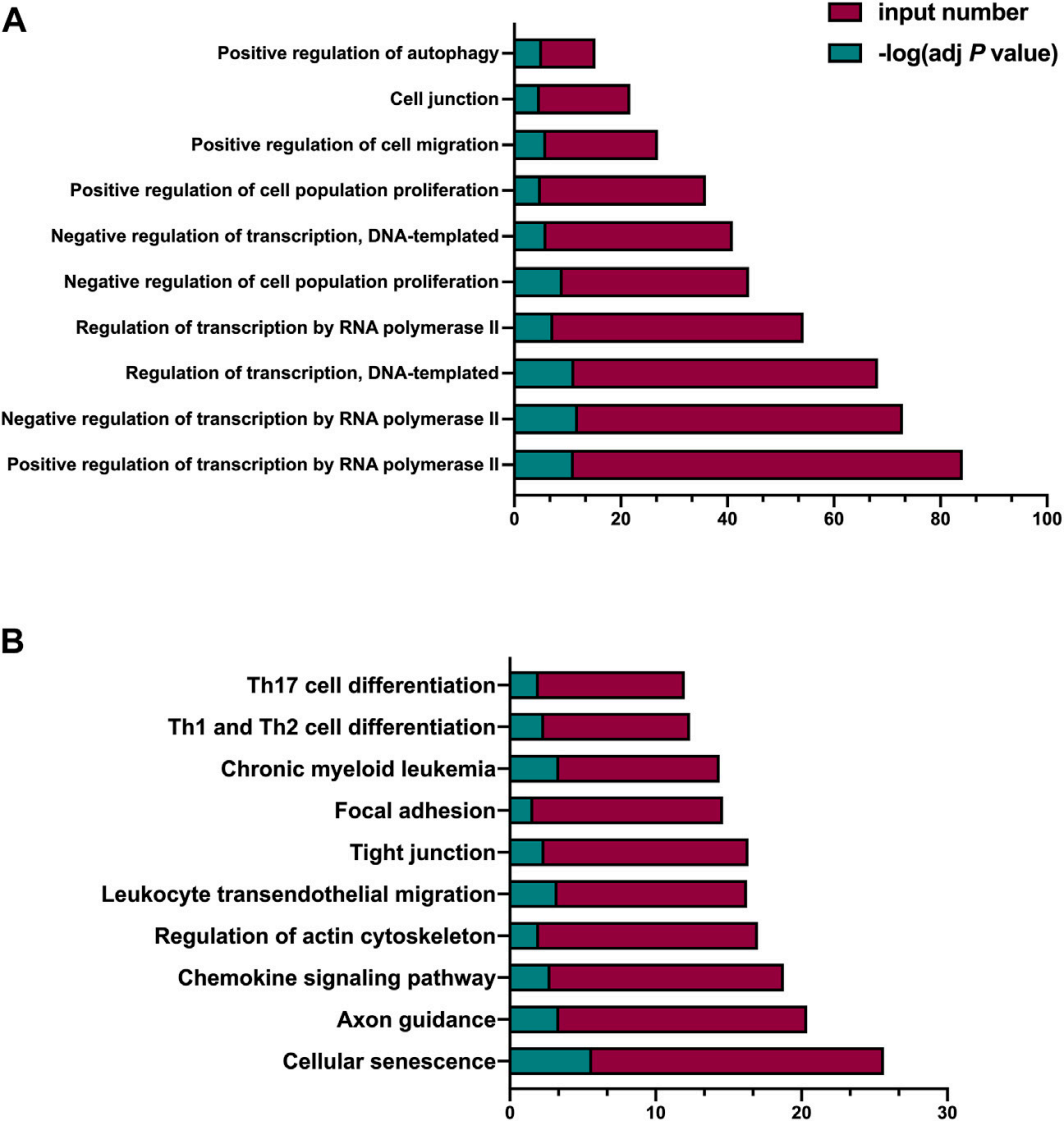
Bioinformatic analysis of the miRNA-23a, miRNA-99a, and lncRNA H19 in the noncoding RNA score. **(A)** GO analysis of the variables in the noncoding RNA score. **(B)** KEGG analysis of the variables in the noncoding RNA score. adj *p* value indicated the FDR-adjusted *p* value; input number indicated the number of enriched targeted mRNAs; GO, Gene Ontology; KEGG, Kyoto Encyclopedia of Genes and Genomes.

To investigate the possible enriched biological processes of the noncoding RNA score, we performed bioinformatic analysis of the miRNA-23a, miRNA-99a, and lncRNA H19 in the noncoding RNA score, the top ten significantly enriched biological processes included “Positive regulation of transcription by RNA polymerase II”, “Negative regulation of transcription by RNA polymerase II”, “Regulation of transcription, DNA-templated”, “Regulation of transcription by RNA polymerase II”, “Negative regulation of cell population proliferation”, “Negative regulation of transcription, DNA-templated”, “Positive regulation of cell population proliferation”, “Positive regulation of cell migration”, “Cell junction” and “Positive regulation of autophagy” (Figure 2A); and the top ten significantly enriched KEGG pathways included “Cellular senescence”, “Axon guidance”, “Chemokine signaling pathway”, “Regulation of actin cytoskeleton”, “Leukocyte transendothelial migration”, “Tight junction”, “Focal adhesion”, “Chronic myeloid leukemia”, “Th1 and Th2 cell differentiation”, and “Th17 cell differentiation” (Figure 2B).

## Discussion

In the present study, we demonstrate an association between the noncoding RNAs of circulating neutrophils and the outcomes of AIS patients received rtPA treatment. A higher noncoding RNA score composed of miRNA-23a, miRNA-99a, and lncRNA H19 is related to sICH after rtPA treatment and a poor outcome at 3 months. Of note, after adjustment for confounders, we find that the noncoding RNA score is an independent predictor for the poor outcome at 3 months and sICH after rtPA treatment, with an additional 2.16-fold risk to have a poor outcome and an additional 5.25-fold risk to develop sICH for noncoding RNA score ≥ −0.336, respectively. Additionally, the noncoding RNA score adds prognostic value to the clinical model for predicting the poor outcome and sICH after rtPA administration. Particularly, the noncoding RNA score is positively correlated with the infarct volume after rtPA treatment, and a lower noncoding RNA score is associated with symptom improvement at 7 days after rtPA treatment.

Noncoding RNA is one of the most promising biomarkers for early diagnosis, risk stratification, and prognosis of stroke (Kadir et al., 2022). A large clinical study found that 7 miRNAs were significantly associated with cerebrovascular disorder and a model of 3 miRNAs combination could predict the risk of stroke (Sonoda et al., 2019). Another clinical study involving 22 AIS patients and 6 AIS patients received treatment demonstrated that miRNAs altered during the onset and treatment and possessed potential value for predicting symptom progression after stroke (Abe et al., 2020). In line with these studies, we observe that a higher neutrophilic miRNA-23a level is associated with moderate-to-severe stroke on admission and symptom improvement at 24 h after rtPA treatment, and the noncoding RNA score composed of miRNA-23a, miRNA-99a, and lncRNA H19 is associated with the infarct volume and symptom progression at 7 days after rtPA treatment.

A prospective cohort study involved 84 AIS patients received thrombolysis or bridging therapy found three circulating miRNAs detected at 24 h after treatment were associated with the unfavorable outcomes at 3 months (He et al., 2019). Consistently, we demonstrate that a higher noncoding RNA score composed of miRNA-23a, miRNA-99a, and lncRNA H19 is associated with a poor outcome at 3 months and the incidence of sICH after rtPA treatment. Moreover, our study adds the evidence that the neutrophilic noncoding RNA score before thrombolysis possesses predictive value to identify patients with worse outcomes after rtPA treatment. Furthermore, we also compare the predictive models with and without the noncoding RNA score and find that the addition of noncoding RNA score improves the prognostic efficiency of the clinical models for sICH and functional outcome at 3 months after thrombolysis. In clinical practice, a patient presented with a higher noncoding RNA score before rtPA treatment may need timely endovascular recanalization and more intensive medical care.

Noncoding RNA targets multiple genes and proteins and plays a key role in various aspects of the complicated pathological processes of ischemic stroke (Kaur et al., 2018), which make noncoding RNA meets the demand of the comprehensive evaluation of multiple targets for clinical decision-making of thrombolysis. Bioinformatic analysis of neutrophilic miRNA-23a, miRNA-99a, and lncRNA H19 in the noncoding RNA score suggests that they are significantly enriched in the biological processes including transcription, cell proliferation, cell migration, cell junction, and cell autophagy; and enriched in some crucial signaling pathways. The action of these noncoding RNAs on cell proliferation and the interaction of these noncoding RNAs in “Chronic myeloid leukemia”, which is consistent with the knowledge that surging neutrophil counts after stroke is responsible for physical obstruction induced no-reflow (El Amki et al., 2020) after rtPA treatment; the “Cellular senescence” is reminiscent of the neutrophil extracellular trap induced reperfusion resistance (Peña-Martínez et al., 2019) after rtPA treatment; the action of these noncoding RNAs on cell migration and cell junction and the interaction of these noncoding RNAs in “Chemokine signaling pathway”, “Regulation of actin cytoskeleton”, “Leukocyte transendothelial migration”, “Tight junction”, and “Focal adhesion”, support the role of circulating neutrophils migration during reperfusion and blood-brain barrier damage after thrombolysis (Shi et al., 2021); and “Th1 and Th2 cell differentiation” and “Th17 cell differentiation” point out the possibility that neutrophilic miRNA-23a, miRNA-99a, and lncRNA H19 may take effect on lymphocytes differentiation. Taken together, the bioinformatic analysis underscores the possible pathophysiological processes responsible for the outcomes after thrombolysis that neutrophilic miRNA-23a, miRNA-99a, and lncRNA H19 implicated in, and supports the predictive value of noncoding RNA score before thrombolysis for the outcomes of AIS.

Neutrophils shape the progression of the immune response in the ischemic brain (Jickling et al., 2015), thus participating in determining the severity of neurological impairment and clinical prognosis of AIS patients (Gong et al., 2021). A clinical study involving 846 patients received thrombolysis treatment demonstrated that higher neutrophil counts before thrombolysis were independently associated with the sICH and worse outcome at 3 months, but the mechanisms involved in this effect were unknown (Maestrini et al., 2015). Interestingly, our study hints that miRNA-23a, miRNA-99a, and lncRNA H19 could be the possible mediators responsible for the effect of neutrophils on stroke outcomes after thrombolysis. Preclinical studies found that miR-23a lessened oxidative stress and cerebral ischemia-reperfusion (I/R) injury (Zhao et al., 2014), and miR-23a released by microglia could promote white matter repair in experimental stroke model (Li et al., 2022b); miR-99a prevented neuron apoptosis following cerebral ischemia through regulating cell cycle progression in experimental I/R model (Tao et al., 2015); H19 promoted neuroinflammation by driving M1 microglial polarization (Wang et al., 2017), and promoted leukocyte inflammation by targeting the miR-29b/C1QTNF6 axis in cerebral ischemic injury in experimental stroke model (Li et al., 2022a). We here also observe positive correlation between these three noncoding RNAs, and the best correlation has between found between miR-23a and miR-99a, it may be due to the different effects of microRNA and lncRNA in ischemic stroke, and different mechanisms of action of microRNA and lncRNA. As such, the combinational roles of neutrophilic miRNA-23a, miRNA-99a, and lncRNA H19 on the ischemic stroke are worth further investigation.

The strength of our study is that we generate the pretreatment noncoding RNA score to establish the prediction model for the functional outcome at 3 months and sICH of AIS patients after rtPA treatment, which takes into consideration of the incremental effect of the noncoding RNA interaction network. There are also some limitations in our study. Firstly, the predictive value of the noncoding RNA score for sICH and poor outcome after rtPA treatment needs to be validated in a larger sample size. Secondly, the levels of noncoding RNA may be dynamic, and the temporal changes of neutrophilic noncoding RNAs levels both pre- and post-rtPA treatment in AIS warrant further study.

## Data Availability

The original contributions presented in the study are included in the article/Supplementary Material; further inquiries can be directed to the corresponding authors.

## Supplementary material

Supplementary Figure E1 Flow chart of the study.
