# Supplementary figures and images for: Neutrophilic noncoding RNAs predict outcomes of acute ischemic stroke patients treated with recombinant tissue plasminogen activator
Source: Front Pharmacol. 2022 Oct 6;13:1003806. doi: 10.3389/fphar.2022.1003806 (PMC9582270; doi:10.3389/fphar.2022.1003806)

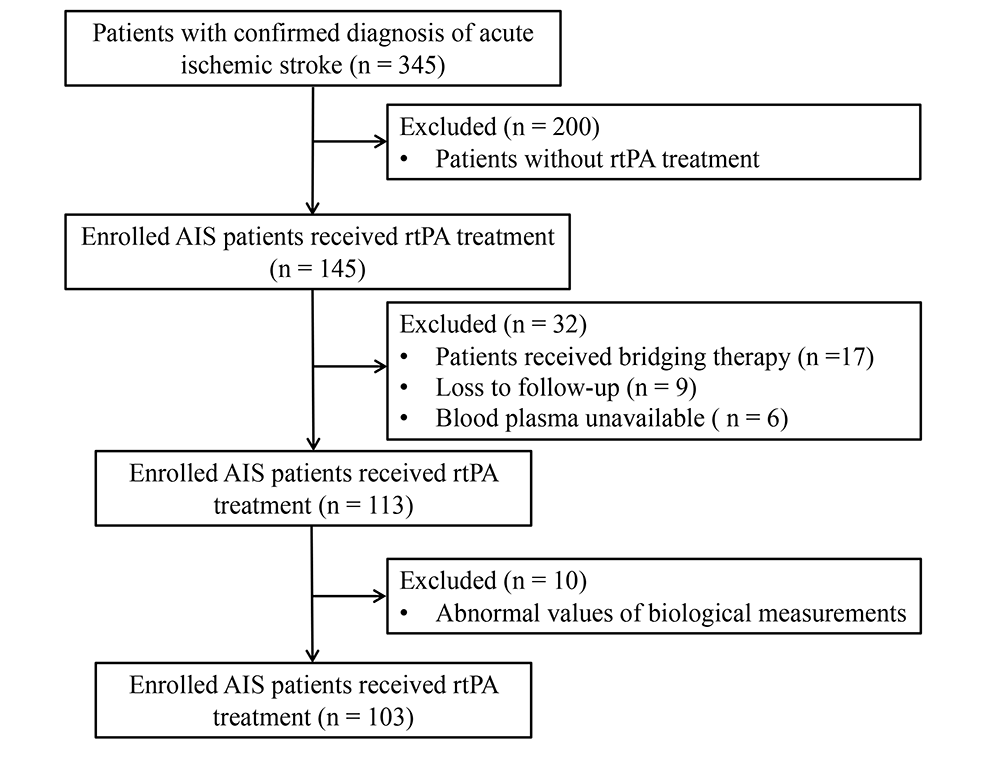

Supplement: Supplementary file 1 [file Image1.tif]
