# Supplementary material for: Neutrophilic noncoding RNAs predict outcomes of acute ischemic stroke patients treated with recombinant tissue plasminogen activator
Source: Front Pharmacol. 2022 Oct 6;13:1003806. doi: 10.3389/fphar.2022.1003806 (PMC9582270; doi:10.3389/fphar.2022.1003806)
Supplement: Supplementary file 2 [file Table2.docx]

**Table e1 Bivariate correlation between noncoding RNAs and the onset-to-treatment time, neutrophil count, and neutrophil-to-lymphocyte ratio in the whole study population (n = 103).**

† *p* < 0.05.

|  | **Onset-to-treatment time, h** | | **Neutrophil count** | | **Neutrophil-to-lymphocyte ratio** | |
| --- | --- | --- | --- | --- | --- | --- |
|  | **ρ** | ***p* Value** | **ρ** | ***p* Value** | **ρ** | ***p* Value** |
| **miR-23a** | -0.01 | 0.909 | 0.02 | 0.847 | 0.04 | 0.670 |
| **miR-193a** | -0.19 | 0.060 | -0.22 † | 0.025 | -0.17 † | 0.048 |
| **miR-128** | -0.19 | 0.057 | -0.18 | 0.071 | -0.22 † | 0.021 |
| **miR-let7a** | -0.19 † | 0.049 | -0.20 † | 0.047 | -0.10 | 0.323 |
| **miR-99a** | -0.20 † | 0.044 | -0.14 | 0.156 | -0.19 | 0.054 |
| **miR-494** | -0.22 † | 0.024 | -0.10 | 0.322 | -0.14 | 0.157 |
| **miR-424** | -0.07 | 0.502 | 0.17 | 0.094 | 0.19 | 0.053 |
| **Lnc H19** | 0.10 | 0.334 | -0.01 | 0.964 | 0.11 | 0.268 |

|  | **Youden index, %** | **Cutoff point** | | **Sensitivity, %** | **Specificity, %** | |
| --- | --- | --- | --- | --- | --- | --- |
| **Noncoding RNA score^#^ for excellent outcome** | 18.2 | | -0.336 | 48.8 | | 69.4 |
| **Noncoding RNA score^#^ for sICH** | 22.3 | | -0.336 | 51.7 | | 77.7 |

**Table e****2 The Youden index of for identifying patients with excellent outcome at 3 months (n = 103).**

# **Noncoding RNA score** = 0.017661997*miR-23a-0.092415475*miR-99a-0.007679703*lnc19-0.209855481.

**Table e3 Baseline characteristics of the whole study population according to the modified Rankin Scale (mRS) at 3 months (n = 103).**

|  | | **Total** | | **Excellent outcome ^a^  Poor outcome** | | | ***p* Value** |
| --- | --- | --- | --- | --- | --- | --- | --- |
|  | | **(N=103)** | | **(N=62)** | **(N=41)** | |  |
| **Demographic characteristics** | |  | |  |  | |  |
| **Age, y, mean (SD)** | | 61.9 (12.7) | | 60.0 (12.1) | 64.6 (13.1) | | 0.071 † |
| **Female sex (%)** | | 22 (21.4) | | 14 (22.6) | 8 (19.5) | | 0.899 |
| **BMI, kg/m^2^, median [IQR]** | | 25.6 [25.1, 26.2] | | 25.6 [25.0, 26.2] | 25.6 [25.6, 26.1] | | 0.648 |
| **Medical history** | |  | |  |  | |  |
| **Hypertension** | | 71 (68.9) | | 31 (33.9) | 40 (73.2) | | 0.190 † |
| **Diabetes mellitus** | | 29 (28.2) | | 16 (25.8) | 13 (31.7) | | 0.669 |
| **Hyperlipemia** | | 23 (22.3) | | 11 (17.7) | 12 (29.3) | | 0.257 |
| **Coronary heart disease** | | 19 (18.4) | | 12 (19.4) | 7 (17.1) | | 0.174 † |
| **Atrial Fibrillation** | | 17 (16.5) | | 6 (10.1) | 11 (23.0) | | 0.147 † |
| **Recurrent stroke** | | 30 (29.1) | | 14 (22.6) | 16 (39.0) | | 0.215 |
| **Smoking habit** | | 39 (37.9) | | 23 (37.1) | 16 (39.0) | | - |
| **Clinical and laboratory findings** | | | | | | | |
| **Systolic blood pressure, mm Hg** | | 150.0 [140.0, 166.0] | | 153.0 [140.0, 167.5] | 149.0 [140.0, 165.0] | | 0.669 |
| **Diastolic blood pressure, mm Hg** | | 83.0 [74.5, 92.0] | | 82.5 [73.8, 92.0] | 86.0 [75.0, 92.0] | | 0.681 |
| **Serum glucose, mmol/L** | | 7.1 [5.9, 10.5] | | 7.0 [5.8, 9.9] | 7.2 [6.1, 10.6] | | 0.320 |
| **Neutrophils, ×1,000/mm^3^** | | 4.8 [3.7, 6.3] | | 4.8 [3.8, 6.2] | 4.4 [3.5, 6.5] | | 0.450 |
| **NLR** | | 1.6 [1.2, 2.2] | | 1.9 [1.2, 2.2] | 1.5 [1.2, 2.2] | | 0.166 † |
| **Platelet count, ×1,000/mm^3^** | | 2.8 [2.0, 4.6] | | 2.6 [2.0, 4.2] | 2.9 [2.0, 5.1] | | 0.440 |
| **TG,** **mmo****l/L** | | 1.7 [1.1, 2.7] | | 1.7 [1.0, 2.8] | 1.7 [1.2, 2.5] | | 0.809 |
| **TC, mmol/L** | | 4.6 [3.8, 5.4] | | 4.9 [3.8, 5.8] | 4.4 [3.6, 4.9] | | 0.043 † |
| **HDL, mmol/L** | | 1.2 [1.0, 1.4] | | 1.2 [1.0, 1.4] | 1.2 [1.1, 1.4] | | 0.390 |
| **LDL, mmol/L** | | 2.6 [2.1, 3.4] | | 2.7 [2.2, 3.7] | 2.5 [2.0, 3.1] | | 0.218 |
| **Stroke characteristics and treatment** | |  | |  |  | |  |
| **Admission NIHSS score** | | 5.0 [3.0, 10.0] | | 4.5 [2.2, 6.0] | 9.0 [5.0, 14.0] | | <0.001 † |
| **Onset-to-treatment time, h** | | 2.2 [1.2, 3.2] | | 2.0 [1.1, 2.9] | 2.7 [1.4, 3.3] | | 0.042 † |
| **Bridging therapy** | | - | | - | - | | - |
| **Stroke etiology (TOAST), n (%)** | | | | | |  | |
| **Large artery atherosclerosis** | 63 (61.2) | | 39 (62.9) | | 24 (58.5) | 0.811 | |
| **Small vessel occlusion** | 17 (16.5) | | 11 (19.8) | | 6 (12.4) | 0.220 | |
| **Cardioembolic** | 12 (11.7) | | 4 (6.5) | | 8 (19.5) | 0.088 † | |
| **Other determined** | 4 (3.9) | | 1 (1.6) | | 3 (7.3) | 0.344 | |
| **Undetermined** | 7 (6.8) | | 4 (6.5) | | 3 (7.3) | - | |
| **Posterior circulation stroke** | 10 (9.7) | | 4 (7.3) | | 6 (12.5) | 0.575 | |
| **Noncoding RNA measures** | | | | | | | |
| **miR-23a** | | 2.5 [1.1, 5.7] | | 2.7 [1.2, 5.4] | 1.8 [0.9, 5.9] | | 0.716 |
| **miR-193a** | | 2.0 [1.2, 3.4] | | 1.8 [1.1, 3.4] | 2.5 [1.3, 3.3] | | 0.571 |
| **miR-128** | | 3.7 [1.5, 7.1] | | 2.9 [1.6, 8.2] | 3.9 [1.5, 6.4] | | 0.824 |
| **miR-let-7a** | | 3.9 [2.0, 8.4] | | 3.4 [1.7, 8.5] | 4.7 [2.4, 7.7] | | 0.736 |
| **miR-99a** | | 2.3 [1.2, 4.8] | | 2.3 [1.3, 5.4] | 2.5 [1.0, 4.0] | | 0.451 |
| **miR-494** | | 1.2 [0.7, 2.4] | | 1.2 [0.7, 2.4] | 1.1 [0.6, 2.2] | | 0.531 |
| **miR-424** | | 3.6 [2.5, 5.7] | | 3.7 [2.5, 5.5] | 3.5 [2.6, 5.8] | | 0.782 |
| **Lnc H19** | | 1.7 [1.1, 2.8] | | 1.7 [1.1, 2.5] | 1.6 [1.1, 3.0] | | 0.882 |

† *p* < 0.2

**Table e4 Comparisons of Noncoding RNA score according to the** **symptom improvement at 7d after rtPA treatment (n = 103).**

|  | **Total** | **Symptom improvement at 7d*** | | ***p* Value** |
| --- | --- | --- | --- | --- |
|  | **(N= 103)** | **(No, N= 65)** | **(Yes, N= 38)** |  |
| **Noncoding RNA score** | -0.4 [-0.6, -0.3] | -0.3 [-0.5, -0.3] | -0.4 [-0.6, -0.3] | 0.036 |
| **Noncoding RNA score ≥** -0.336 | 39 (38.6) | 29 (46.8) | 10 (26.3) | 0.048 |

*****7dΔNIHSS (NIHSSbaseline - NIHSS7d) ≥ 4 was considered as an improvement of symptoms at 7 d.

**Table e5 Comparisons of** **Noncoding RNA score according to the** **symptom improvement at 24h after rtPA treatment (n = 103).**

|  | **Total** | **Symptom improvement at 24h*** | | ***p* Value** |
| --- | --- | --- | --- | --- |
|  | **(N= 103)** | **(No, N= 79)** | **(Yes, N= 24)** |  |
| **Noncoding RNA score** | -0.4 [-0.6, -0.3] | -0.4 [-0.6, -0.3] | -0.4 [-0.5, -0.3] | 0.861 |
| **Noncoding RNA score ≥** -0.336 | 39 (37.9) | 31 (39.2) | 8 (33.3) | 0.871 |

*24hΔNIHSS (NIHSSbaseline – NIHSS24hours) ≥ 4 was considered as an improvement of symptoms at 24h.
